# Undergraduate research implementation in physiotherapy: a hands-on and real experience of a randomised controlled trial

**DOI:** 10.1186/s12909-023-04716-0

**Published:** 2023-10-06

**Authors:** Igor Sancho, Maialen Araolaza-Arrieta, Iker Villanueva-Ruiz, Ane Arbillaga-Etxarri

**Affiliations:** Deusto Physical TherapIker, Physical Therapy Department, Faculty of Health Sciences, University of Deusto, Donostia-San Sebastián, Spain

**Keywords:** Physiotherapy, Undergraduate research, Evidence-based practice.

## Abstract

**Background:**

Evidence-based practice (EBP) is the gold standard approach in physiotherapy, and it is essential that students are aware that it is the appropriate way to provide the patient with the best possible treatment. Undergraduate research (UR) can positively influence learning outcomes and research competencies related to EBP compared to traditional methods of higher education. The aim of this study was to evaluate the effect of implementing a research-based activity (i.e., active participation in a randomised controlled trial [RCT]) in the UR programme on the learning and acquisition of research methodology-related competencies by first-year physiotherapy students.

**Methods:**

Students in the first academic year of the Bachelor´s Degree in Physiotherapy of University of Deusto (Donostia-San Sebastian, Spain) who were enrolled in the subject ‘Introduction to Research Methodology’ were invited to take part in a real RCT which consisted of three groups: intervention, placebo, and control group. While the RCT was carried out, researchers and/or participants roles were combined among students during the semester. At the end, a questionnaire that included open and closed questions was used to evaluate the effectiveness of the UR strategies used in students´ acquisition of theoretical knowledge, research competencies, self-efficacy of RCT skills and procedures. Lecturers also completed the questionnaire to evaluate their experience.

**Results:**

From the 114 students enrolled in the subject, 102 participated in the RCT and 110 answered the final questionnaire. Regarding the development of research competencies, UR had a positive or very positive impact on critical thinking (67% and 18%, respectively) and in the assessment of methodological quality (66% and 23%, respectively). Furthermore, most students reported that the implementation of the RCT facilitated their knowledge of placebo, detection of bias, development of critical thinking and a better understanding of methodological issues in research. Lecturers reported an additional burden that was difficult to reconcile with daily duties.

**Conclusion:**

The novel UR program provided students with a new opportunity to improve their knowledge of RCT procedures, thus making the learning process more meaningful. Therefore, ways of teaching and learning focused on improving research and inquiry attitudes should be considered and integrated into the health care curriculum, especially in physiotherapy programs, to ensure the transfer of EBP for the provision of the best care.

**Trial registration:**

Australian New Zealand Clinical Registry: ACTRN12622000263796p (14/02/2022).

**Supplementary Information:**

The online version contains supplementary material available at 10.1186/s12909-023-04716-0.

## Background

The development of research skills at higher education is considered a challenge where the institutions and lecturers try to combine educational and research attitudes, while inexperienced and novice students need to develop scientific skills [1]. In this way, research and teaching should be connected as it provides the opportunity for lecturers and researchers, who usually are already involved in the natural process of academic or clinical research, to disseminate, promote and provide scientific knowledge to those students who are just about to develop the basic research skills for their incipient professional future. In this framework, the integration of hands-on research experiences is considered an effective solution to link both profiles [2, 3]. However, in the field of physiotherapy, only a few studies had specifically investigated this relationship [4]. Indeed, rather than analysing which strategies are pedagogically effective for this purpose, most methodologies tend to focus on evidence-based practice (EBP) teaching, due to its relevance in clinical practice [4].

EBP consists in the application of the best scientific evidence in clinical decision-making by integrating clinical experience, incorporating patient values and preferences into the practice of professional patient care [5]. In health science disciplines like physiotherapy, the translation of EBP into practice is vital to adopt a critical stance to provide the best care. Therefore, knowledge of research methodology and critical thinking skills are essential. For example, it is essential to be aware of the issues related to randomized control trials (RCT), namely in terms of methodological biases and trial design features, and how these impact the interpretation of their results and treatment effect estimates. Physiotherapy students should naturally develop and integrate analytical and critical thinking about research to ensure the implementation of the best EBP [6].

However, the term EBP should be clarified since it is considered a general, universal, and gold standard learning outcome for clinical practice rather than a specific education strategy per se (4). In this sense, Bala et al. found that different teaching and learning focused on EBP improved knowledge and changed behaviour across a diverse range of teaching modalities and health students. Nevertheless, most of the studies were considered as critically low quality. Therefore, the most effective teaching strategies to promote the use of EBP in clinical practice are uncertain (7–9). In this context, one study showed that the meaning of EBP processes or principles are not well understood by undergraduate health students (10). Hence, the approach of linking research and teaching in higher education seems to require a holistic educational environment rather than a learning outcome.Undergraduate research (UR) can positively influence learning outcomes and facilitate the acquisition of research competencies related to EBP when compared to traditional methods in higher education (11–14). Undergraduate research consists of the application of a battery of teaching and learning strategies that aim to aid the student in gaining research-related knowledge and skills. This is accomplished by providing students with opportunities to acquire research skills and apply theoretical contents in real practice, for example, by involving students in a partial or full research project under the lecturer’s supervision [2]. This activity facilitates the appropriation and construction of knowledge through practice, collaborative learning, experimentation, and critical thinking. Active participation through first-hand experience helps students learn about and foster interest in the disciplines developed [15]. Consequently, UR provides a unique and special environment that merges scientific and educational procedures at the same time.

Evidence supports that UR implementation increases motivation and develops an investigative attitude and vital general skills in students [16]. Recent scoping reviews have been carried out among students involved in rehabilitation issues including physiotherapy and/or occupational therapy programmes [17–19]. However, the implementation of UR across healthcare programs (i.e., physiotherapy) is still limited [20]. The aim of this study was to evaluate the effect of the implementation of a research-based activity (i.e., active participation in an RCT) in the UR program on the learning and acquisition of competences related to research methodology by first-year physiotherapy students.

## Methods

A real RCT where students could take part as researchers and/or participants as the main UR strategy was undertaken. The aim of the RCT was to evaluate the effectiveness of a superficial neuromodulation device developed by an external private company (© 2020 Irmoki). However, the true aim of the study reported here, to which students taking part were blind, was to evaluate the effect of the implementation of UR (through a teaching and learning strategy that included the design and active participation of students in an RCT) as described earlier.

First-year students of the Bachelor’s Degree in Physiotherapy and the Bachelor’s double Degree in Physiotherapy and Physical Activity and Sport Sciences, who were enrolled in the subject ‘Introduction to Research Methodology’ at University of Deusto during the course 2021-22 were invited to participate in the study. The lectures are taught by two lecturers in three different languages (Basque, Spanish, and English). One lecturer was in charge of one group (the Basque group), whereas the other lecturer led the Spanish and English groups. The number of students for both lecturers was very similar, and they were strictly coordinated to teach in parallel, sharing content, teaching methods, schedule, and evaluation system. Indeed, the subject of “Biostatistics” was also shared between both of them. Finally, they can be considered as active researchers (publishing articles, leading projects and attending congresses) in their background of musculoskeletal and respiratory physiotherapy area, respectively.

It is worth mentioning that the University of Deusto implemented the Degree in Physiotherapy in the academic year 2020–2021. The emerging academic frame allowed the early integration of UR thanks to a curriculum designed according to guidelines set by experts [3], that also included other innovative educational methodologies such as simulation or problem-based learning. ‘Introduction to Research Methodology’ is taught in the second semester of the first year with the aim of seeking across-effect and connecting subsequent courses of the Degree. In this sense, the subject is complemented by other syllabus oriented at EBP in the 2nd, 3rd, and 4th years, which includes research-related activities such as literature reviews, debates, attendance to scientific conferences, or clinical practices in the emerging research group, and the final year dissertation.

For the sake of clarity, two types of procedures have been distinguished: first, those related to RCT procedures, and then those related to UR programme implementation.

### Procedures related to RCT

#### Study design and registration

This RCT was designed following the Consolidated Standard of Reporting Trials for Controlled Studies (CONSORT) statement, registered with the Australian New Zealand Clinical Registry (ACTRN12622000263796p, 14/02/2022) and approved by the Research Ethics Committee of the University of Deusto (ETK-21/21–22). Details about the RCT are presented in Supplementary material A section.

#### Participants

All students (n = 114) enrolled in the subject were invited to participate and informed about the RCT including potential effects and demands of the intervention. Four students refused to participate in the study and eight students dropped out during the 14 weeks due to failure to complete the weekly questionnaire, general discomfort associated with the device, and an ankle sprain. In the end, 102 students finally completed the RCT.

#### Methods

**Fig. 1:**
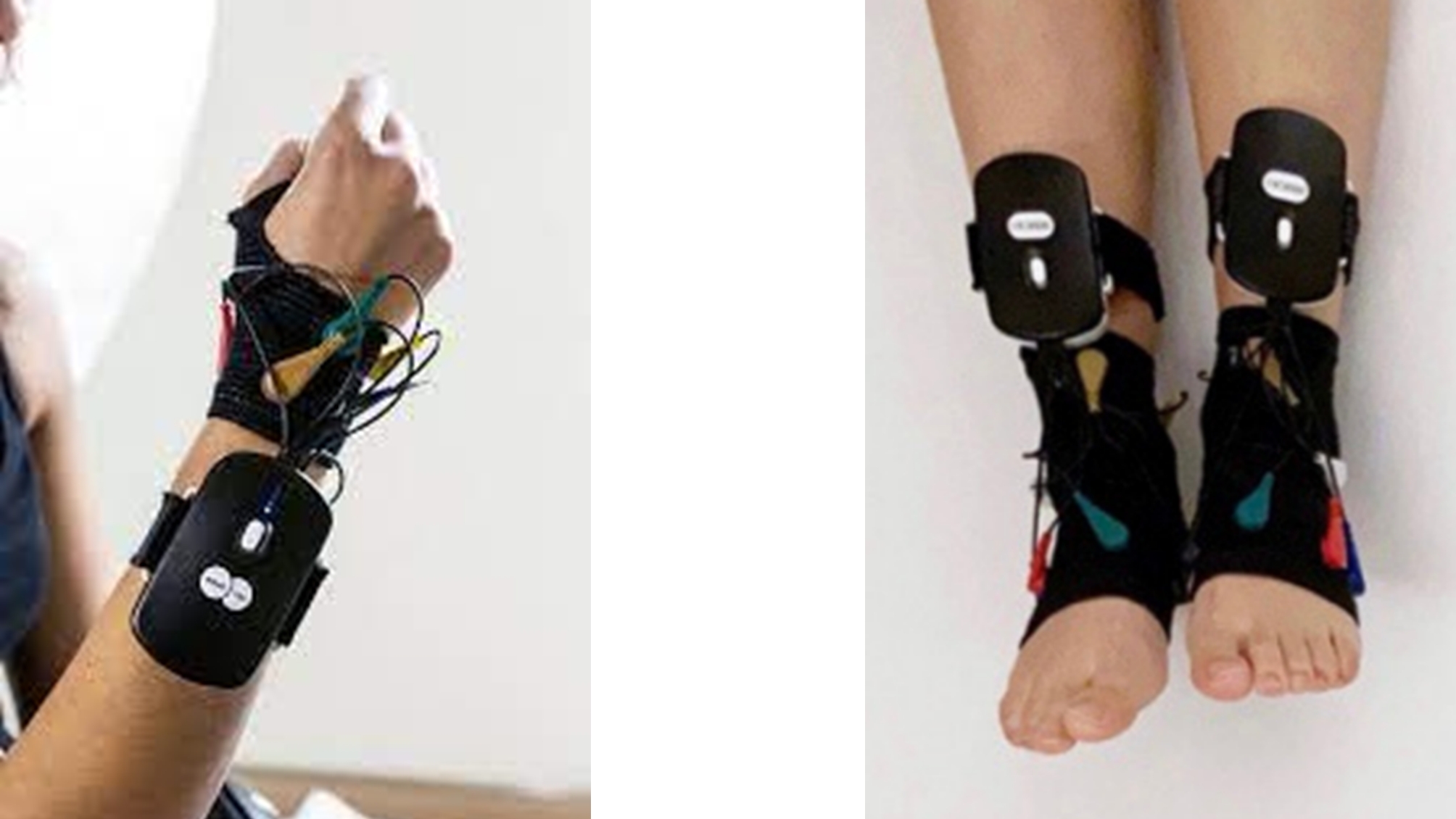
Example of neuromodulation device placement of the intervention and placebo groups. Note: image provided by Irmoki company

Participants were randomly allocated to three groups (intervention, placebo, and control). Students allocated into the intervention group wore a superficial neuromodulation device (© 2020 Irmoki) while students remained seated at rest following lectures at University. The device consists of 4 wireless receptors to be placed on the distal third of the limbs by means of gloves and anklets and controlled through Bluetooth technology by a smartphone app (Fig. 1). The app remotely activates the device which emits rectangular biphasic and monophasic galvanic electrical impulses of very low frequency (0.5–14 Hz) coordinated through 28 electrodes that aim to modulate the autonomic nervous system. Students in the placebo group were blinded by wearing the device in the same way as the intervention group, but without being active (light was switched on but the device did not emit the electrical current). The control group did not receive any intervention.

#### Data collection and analysis

Sociodemographical data were collected at baseline. Outcomes were gathered by means of an online questionnaire at baseline and after weeks 2, 4, 6, and 8 which retrieved data about fatigue, sleep quality, muscular soreness, stress level, and mood. In addition, participants in the intervention and placebo groups were asked about the expectation towards the intervention and the perceived potential alterations.

### Procedures related to the implementation of the UR programme

#### Participants

From 114 students enrolled in the subject, ergo involved in the implementation of the UR teaching strategies for 14 weeks, the same four students who refused to participate on the RCT also refused to answer the UR outcome evaluation questionnaires at the end of the semester. Thus, 110 responses were collected. In addition, the two lecturers completed the questionnaire.

#### Methods

**Fig. 2:**
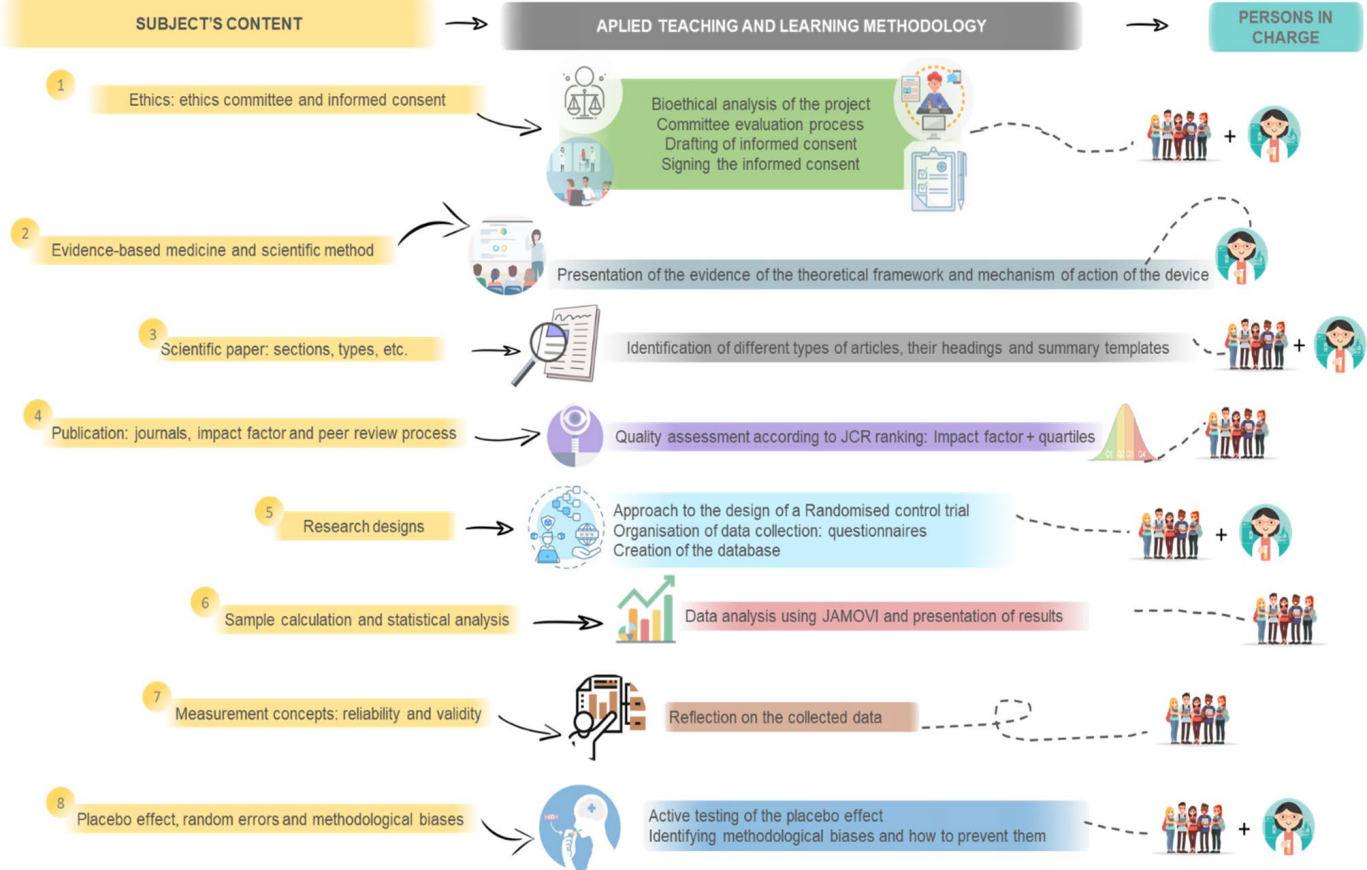
The strategies used during UR methodology implementation

UR strategies were executed according to the subject´s syllabus. These were based, combined, and integrated on the theoretical topics described in Fig. 2. The key research-based strategy was to engage students (as participants and/or researchers) in the execution of a real RCT. In detail, the students allocated to the RCT control group were assigned, under the supervision of the lecturers, the role of managing the delivery and collection of the devices, as well as the adherence to the intervention and the sending and completion of the questionnaires. The students were involved and progressively integrated to work through practical hands-on experiences, in parallel with the theoretical content of the subject. The practical hands-on experiences were: (1) The assessment of the ethical aspects and consent-informed writing was carried out as an experiential activity with the aim of improving the analysis and understanding in depth of the meaning of signing a consent form; (2) In order to contextualise the EBP approach, different papers were used by the lecturers during the presentation to introduce the theoretical framework and the mechanism of action of the device. Likewise, the RCT was used as the thread of the scientific method; (3) The theoretical content of the structure and typology of the papers was introduced through the analysis of the evidence used in the RCT presentation; (4) In order to promote the trend of filtering and ranking the evidence, the quality analysis of the journals was carried out in relation to Journal Citation Reports ranking system; (5) The established RCT design aimed to foster the contrast in depth of the different trials modalities and their limitations in terms of randomisation, blinding, and the presence of a control group; (6) The limited sample size of the RCT prompted reflection about the importance on the previous sample size calculation. Indeed, the lecturers distributed the RCT database with the aim of promoting an experiential preliminary statistical analysis in small groups under the supervision of the lecturers, using the knowledge and skills acquired in the previous subject of “Biostatistics”. In addition, the students were able to carry out a self-assessment by comparing their analysis with the results of the lecturers in a collaborative way; (7) With the aim of fostering the capacity for critical methodological analysis validity, reliability, and especially the bias (such as, selection bias, performance bias, attrition bias, etc.) were thoroughly analysed; (8) As a final activity, the placebo group was revealed to promote critical thinking on the impact of suggestion, expectation, sample contamination, etc.

The RCT, the implementation of UR strategies, and the evaluation were led by the two main lecturers of the subject. In addition, other lecturers from other subjects collaborated exclusively with the task of delivering and collecting the devices. In this sense, they also remained blinded to randomization and study information.

#### Data collection and outcome measures

At the end of the semester, an ad hoc questionnaire completed by participants in the study collected several outcomes focused on four dimensions: (a) the integration of research knowledge related to the theoretical contents; (b) the development of research competencies; (c) the level of self-efficacy about the skills trained; and (d) the RCT procedures. The questions related to the first two dimensions were based on the validated version of the *“Cuestionario de Efectividad del Uso de Metodologías de Participación Activa (CEMPA)”* (21) which measures the perception on the effectiveness of using active participation methodologies. The self-efficacy questions were adapted from the *“Cuestionario de estrategias profundas de aprendizaje”* validated by Panadero et al. [22]. The questionnaire is presented in the Supplementary material B section. Finally, participants and the two lecturers answered an open question to highlight specific elements during the learning process.

### Statistical analysis

Statistical analysis was conducted using Jamovi (v. 1.6.23, The Jamovi Project, Sydney, Australia). Descriptive statistics were calculated with proportions and frequencies and the distribution of the investigated variables was presented with bar diagrams.

## Results

**Fig. 3:**
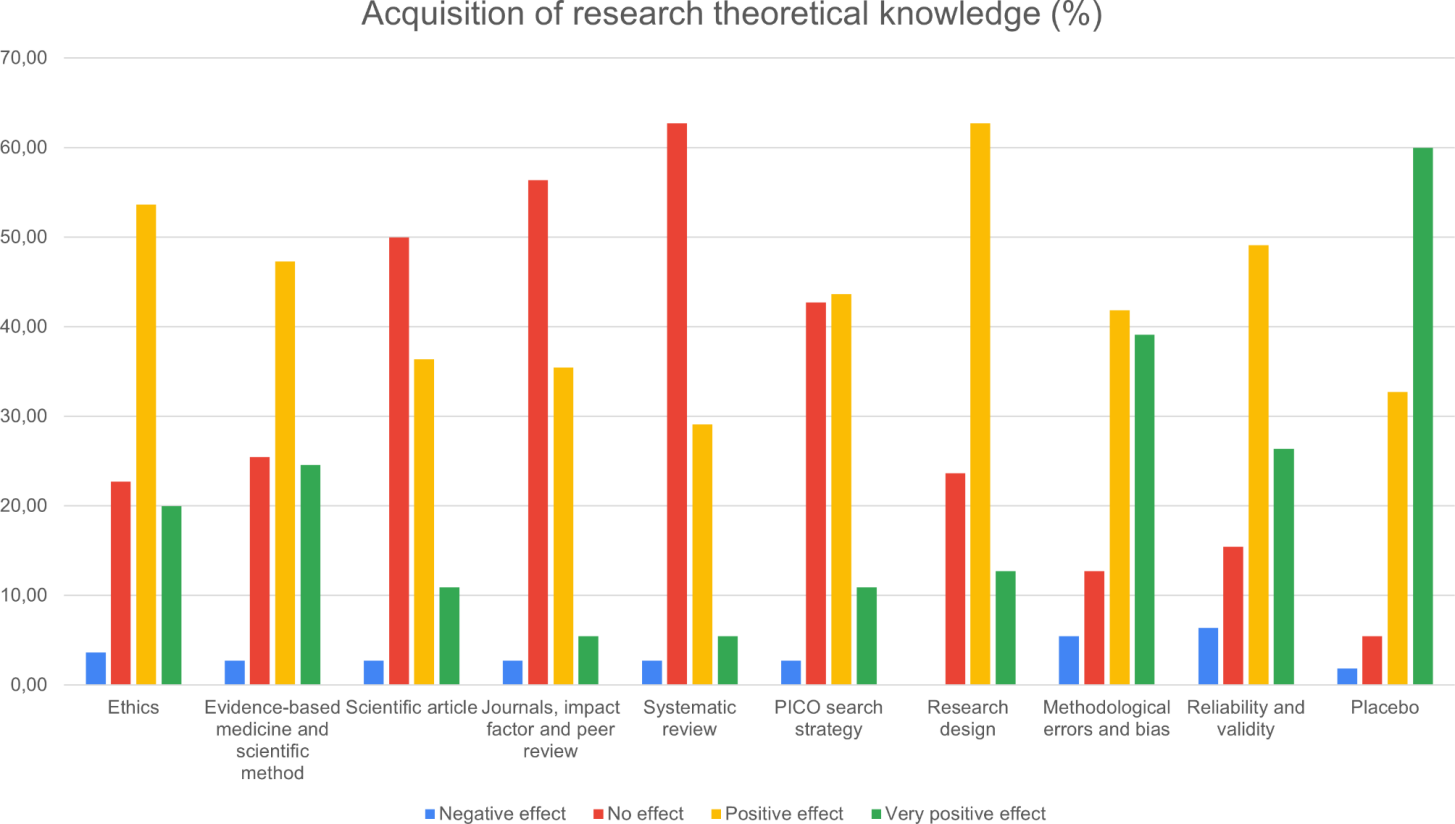
Results of the questionnaire: effect on integration of research knowledge related to the theoretical contents

**Fig. 4:**
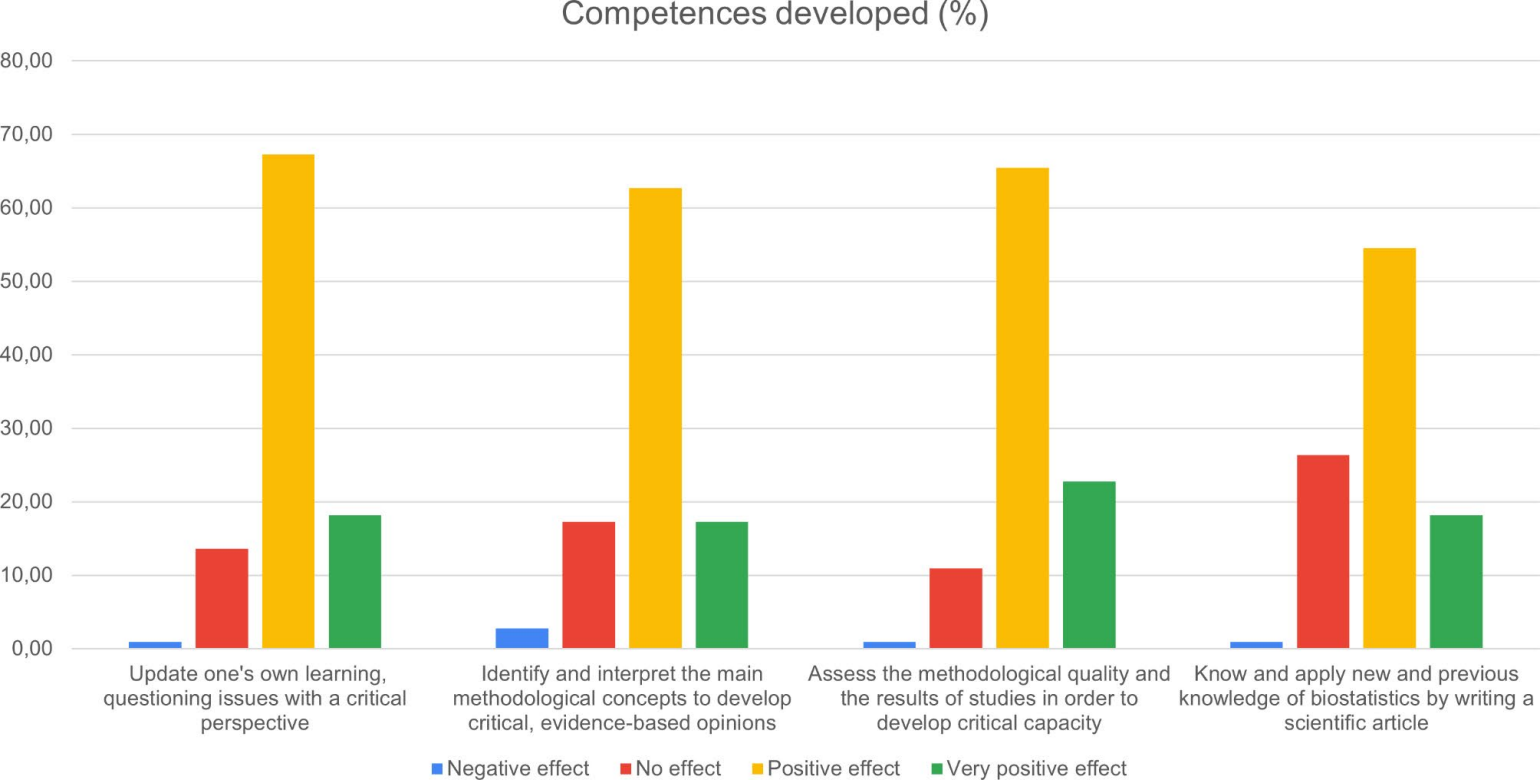
Results of the questionnaire: effect on the development of research competences

**Fig. 5:**
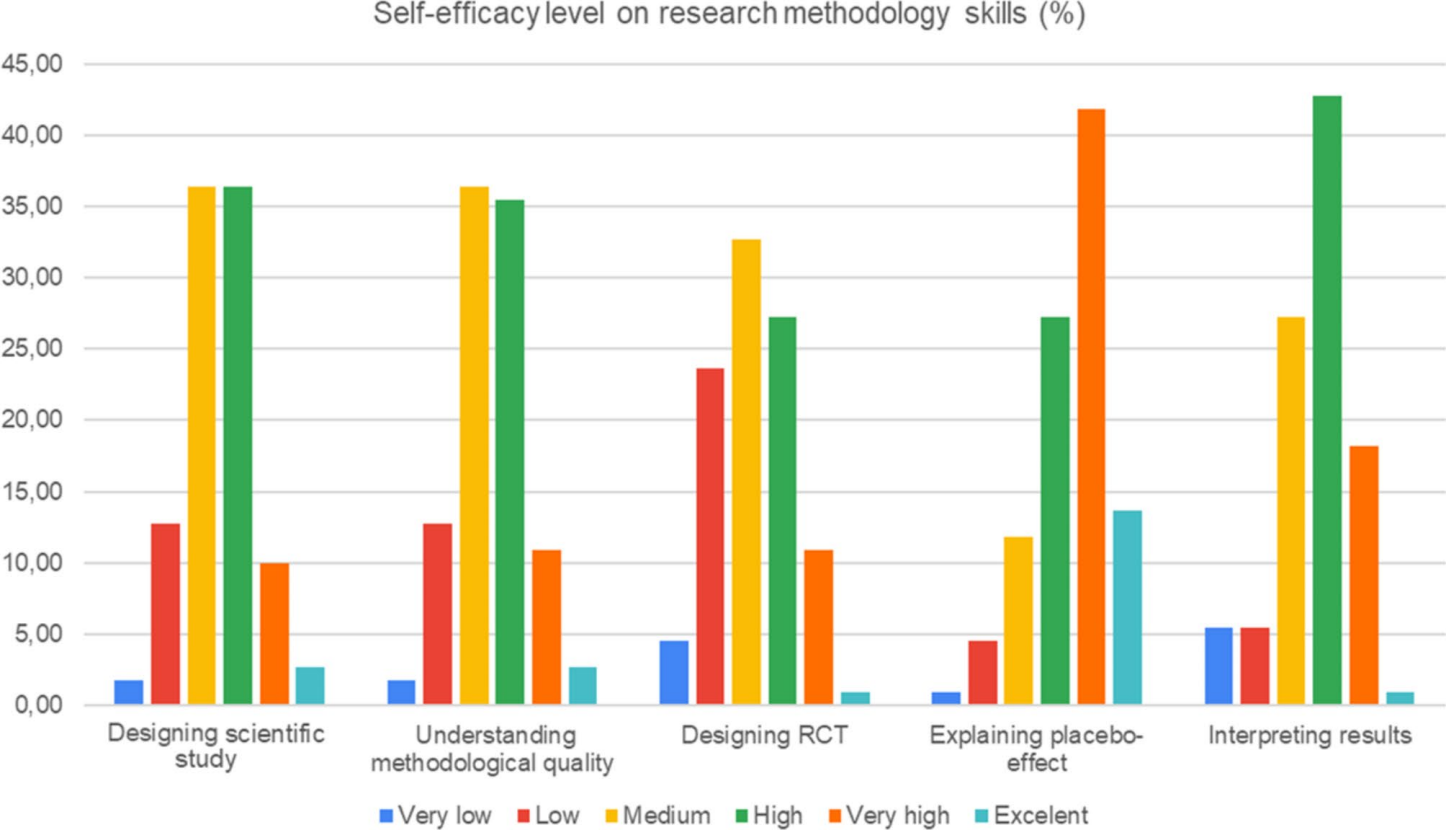
Results of the questionnaire: effect on self-efficacy about the skills trained

**Fig. 6:**
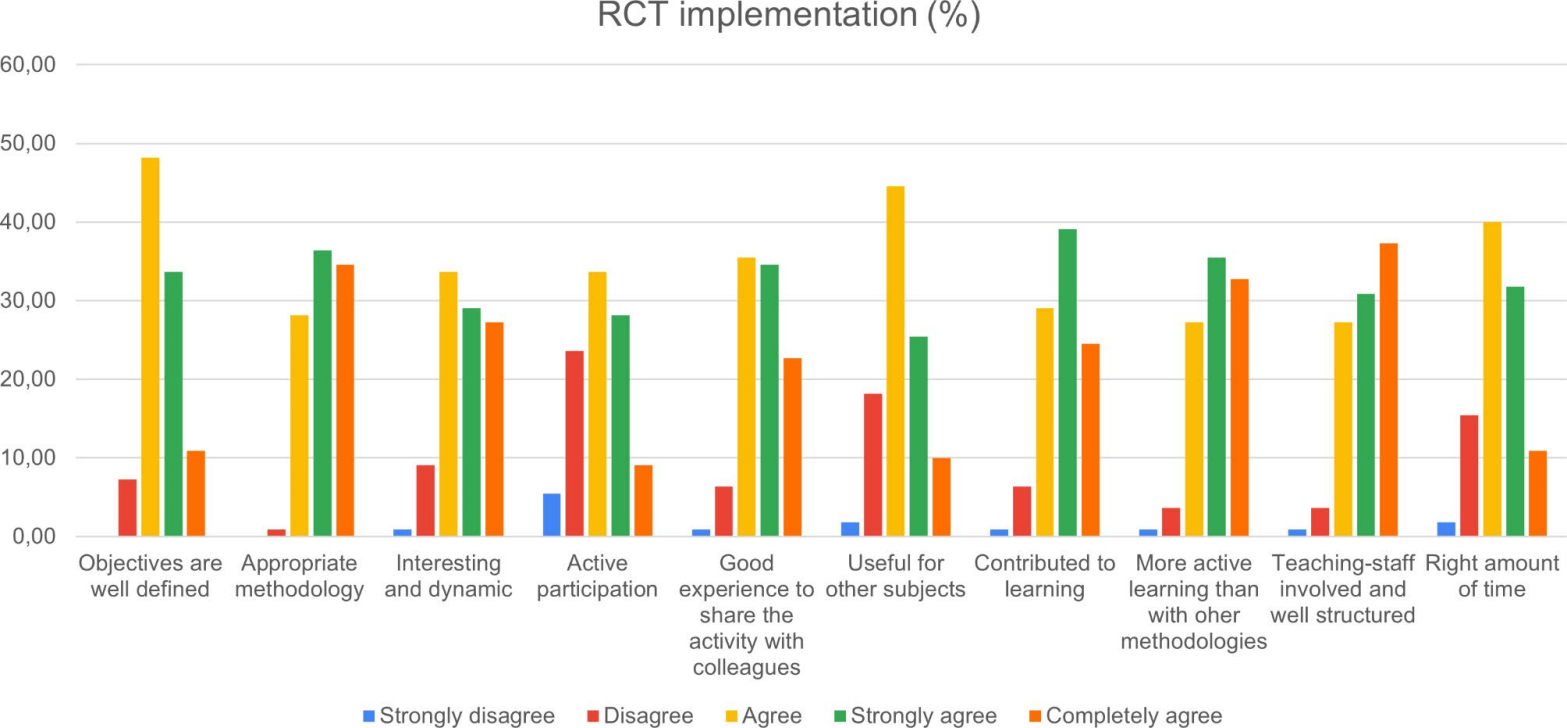
Results of the questionnaire: Issues related to RCT implementation the aim of seeking across effect

110 participants (sex: 48 M/54F; and age: 19.5 (1.63) years) completed the questionnaire about the impact of the UR teaching strategies. Figures 3, 4, 5 and 6 report on the quantitative data provided by the questionnaire. The effect of UR on the acquisition of research knowledge related to theoretical content was very positive (Fig. 3) and especially topics related to the placebo effect, methodological bias, trial design and types of trials, and the concepts of reliability and validity were better adjusted. The smallest effect was found on scientific publications, journal rankings, and PICO strategy.

Regarding the development of research competencies, most students reported that UR had a positive or very positive impact on critical thinking (67% and 18%, respectively) and assessment of methodological quality (66% and 23%, respectively) (Fig. 4).

Self-efficacy about the skills trained was highly rated, and skills that benefited the most were the ability to understand and explain the placebo effect, the capacity to read and integrate the results of an RCT, and the ability to identify bias and methodological gaps related to the design and execution of a clinical trial (Fig. 5).

Students evaluated very positively the inclusion of an RCT as an active learning strategy. Aspects that received the best evaluation were the adequacy of the UR strategy with the implementation of the RCT in the subject, the active participation of students during the learning process, and the involvement and structured organisation shown by the teaching staff responsible for the project (Fig. 6).

Answers from the open question are presented in Table 1. Most students reported that their knowledge about the placebo effect had improved through the trial. In addition, the UR strategies were reported to contribute to the development of critical thinking and a better understanding of the strengths and limitations of the research. Finally, UR was perceived as a tool that facilitated the learning process, added dynamism, complemented the subject content to deepen it, and pointed to the need for EBP.

**Table 1:** Answers to the open question

**Students’ answers:**	“The importance of the placebo group in the study, in order to know whether they are truly improved by the intervention or simply by contextual factors.”
	“In my opinion, the RCT implementation has been a support for the subject. It has given us an experience that we have used as an example, and from that example we have been integrating the subject more.”
	“We have learned to be more critical and not to believe all the information we receive about products or devices.”
	“We have learnt how a study is executed, and the consequences of not structuring a good plan to get the best data.”
	“Making Tables 1 and 2 has helped to better understand the type of RCT design.”
	“It has helped to make the subject more dynamic and to see all the characteristics of the research in the first person.”
	“I would emphasise that without being fully aware we have participated in a study and at the same time we are able to interpret the results that have been obtained. In particular, I think that at the end of the study we were much more aware of what could happen to us in the future, specifically with the placebo.”
	“To be directly involved in a study, and to experience it first-hand in order to identify possible drawbacks and limitations.“
	“Although the intervention has been a bit long, it has been very useful for me to learn that people’s comments influence one’s opinions. Hence, it can generate thoughts that the device may be having an effect, when in fact the device is turned off because you belong to the placebo group.”
	“It has helped me to truly realise how important it is to be grounded in EBC; it has also helped me to learn more about how to do it.
	“I think that although the device has not obtained the expected results, at a pedagogical level it has been quite useful to put into practice most of the aspects worked on in class. In particular, the concept of the placebo since the effects of the placebo have been perfectly verified throughout the study.”
	“Probably to be more critical and not to trust everything I am told due to lack of knowledge.”
**Teachers’ answers**:	“Designing the randomised control trial and managing and organising the intervention has become a major effort and has generated burnout syndrome in some of the teachers involved. Not only because the research is complex, but also because the placebo and intervention groups had to be well managed, drop-outs had to be avoided, and all this while students and teachers were attending classes. In addition, the monitoring of the intervention and its administration was demanding”.
	“Discovering the results after finishing the RCT has been exciting. All the students realised how the placebo group was being contaminated with environmental factors.”

On the other hand, negative aspects identified by both lecturers include the excessive duration of the intervention, the extra daily burden of getting students to wear the devices to ensure adherence to the intervention and data collection, and the difficulty of combining it with other daily obligations. However, lecturers reported that confidence in the premise of fostering research awareness, seeing it as the first step on a progressive path within a built UR programme, the support among the academic staff involved and their research experience helped to overcome the obstacle. Lecturers considered that this educational approach helped students to get closer to the reality of research, feel the placebo effect first-hand, identify mistakes and limitations, and propose solutions based on self-criticism. Lecturers also concluded that the activity provided a valuable opportunity for active learning through real experimentation.

## Discussion

The research-based activity framed in a UR programme consisting of the implementation of a real RCT in which students were involved as participants and researchers, supervised by qualified lecturers, showed positive effects on the acquisition of theoretical content, the development of research competences, the level of self-efficacy in research skills and knowledge about RCT procedures. Furthermore, it provided a novel experience for students, making the learning process more meaningful.

Strategies such as journal clubs or conducting a systematic review or meta-analysis are common in UR programs [18]. In fact, in some universities, physiotherapy students conduct primary research projects, or lecturers assigned to a research group assist students conceptualise a project, recruit subjects, and collect data in the last academic year [23–25]. To our knowledge, this is the first study to apply a real RCT as a research-based strategy integrated in an UR programme in physiotherapy higher education.

In our study, most students reported that UR had a positive or very positive impact on critical thinking and assessment of methodological quality. Likewise, student showed that the RCT implementation improved the ability to understand and explain the placebo effect, the capacity to read and integrate the results of an RCT. This means that the students integrated better key knowledge of RCTs, such us how design features, risk of bias or placebo effect may distort results and lead to incorrect conclusions and substantially modulate clinical decision-making in physiotherapy [26]. In this sense, it is well known that an accurate assessment of the methodological quality of trials is essential in the synthesis of study findings in order to appropriately interpret results and effectively guide the clinical decision process [27]. In addition, the development of critical thinking about research methods and the long-term maintenance of the scientific attitude required by the EBP philosophy are fundamental to ensure its implementation [28, 29]. In fact, the practice of EBP remains low among physiotherapists due to several barriers such as lack of resources, training, knowledge, time, and low availability of sources. Therefore, the use of a RCT as a UR strategy at a very early stage in higher education can reinforce academic responsibility and ethical awareness of students and help to overcome those barriers [28, 29]. Also, according to the competencies proposed by the Europe Region World Physiotherapy, the commitment and honesty that comes implicit with the responsibility to keep up to date with the evidence should be ensured and continuously reinforced [30]. Consequently, students as future clinicians, researchers or stakeholders need to master these skills early in order to apply this ability in a natural and integrated way in their daily health care. Therefore, the implementation of first-hand RCT experience in early stages of higher education may be useful for future clinical practice [31], but more research is needed in this area.

According to the EBP approach of the physiotherapy degree curriculum developed at the University of Deusto, the emerging framework enabled the early integration of UR and other innovative educational methods. Moreover, the inclusion of EBP education in early stages is an accreditation requirement for many health professional disciplines [32, 33]. In this sense, these results can confirm that implementation in an early educational setting can ensure the aim of seeking a progressive and cross-cutting effect that helps connect future concepts of the remaining degree courses [34]. Similarly, there should be a progressive development of research attitudes and skills [34], because this early experience is likely to evolve into progressively improved inquiry capabilities with different levels of independence and complexity. In addition, the research skills should be reinforced by other future research activities, adding more sophisticated searches about complex topics and from group to individual or more independent work. In turn, this is likely to allow for more selectivity in other initiatives as well (i.e., selected students invited to participate in lecturer-led research). However, these long-term effects of the early implementation of the RCT experience were not measured and should be investigated through longitudinal studies in the future [34].

The students evaluated very positively the adequacy of UR strategy, the active participation, and the involvement and structured organisation shown by the teaching staff. In this sense, the early and first-hand RCT experience within a subject related to research methodology, where students are actively involved in carrying out real research practical activities, fieldwork and/or act as participants is unique. This experience gave the students the opportunity to practise and become proficient at it, which has been described as an effective method to develop more sophisticated levels of intellectual development [35]. According to Debowski et al. 2006, students with limited active participation in research (i.e., where the lecturer-led activity is focused on teaching research findings or methods) may have a limited theoretical understanding and lack the ability to apply such knowledge to the real-world. To the contrary, by engaging in practical research strategies, students better understand the relevance of research to their professional practice as well as the complications, limitations, gaps, drawbacks, and value of the process [36, 37].

The positive effect related to the integration of the placebo effect was most often mentioned by the students. To the best of our knowledge, this is the only UR strategy that has led to a real experience of the placebo effect among physiotherapy students. When the placebo group was revealed, the students could integrate how different design features of a trial, co-interventions, sample contamination, ambiguity of symptom detection, patient’s or researcher’s biases can have a substantial impact on estimates of treatment effects. Therewith, we consider the impact on the students’ critical appraisal skill was truly outstanding through unusual, exciting, and surprising pedagogical learning.The acquisition of research theoretical knowledge related to scientific articles, journals, systematic review and PICO did not improve as much as other items. We conclude that the design of the RCT encouraged students to focus on the theoretical content that engaged them most actively. Otherwise, the RCT itself is not very related to this type of theoretical content. If the design used was a systematic review instead of an RCT, the opposite would probably be the case with regard to the topics of theoretical content which scored lower.

Finally, the role of lecturers seems to be very important as an active knowledge transfer agent and the person who motivates, supervises, promotes, executes, and develops research linking students as technicians or voluntary participants during research procedures [38]. In this study, lecturers reported a daily extra burden related with RCT procedures which may impact on other academic and management tasks. This should be taken into account by departmental managers to ensure effective, appropriate, and sustainable teaching-research links and experiences [31].

### Limitations

This study has some limitations. A systematic pre-post semester analysis and the lack of a control group of students without UR implementation are the most important. In this sense, a control group was very difficult to achieve because it was practically unfeasible to blind the students to the implementation of the RCT. However, this should be taken into account when interpreting the results obtained. In addition, a long-term view in line with other research and inquiry strategies are required to evaluate the effect on the students’ learning process. Further, increasing the sample size of students should also provide more insights into the teaching and learning capabilities of research. Finally, the lack of validated tools and the use of different points and not well-balanced scales when dimensions were assessed also could have had a negative impact.

## Conclusion

This study presents a novel approach of the framework of UR in the unexplored healthcare discipline. Conducting an RCT is a challenging but valuable, useful, and effective way to integrate research and an inquiring attitude in physiotherapy students. Forms of teaching and learning focused on enhancing research and inquiring attitudes should be considered and integrated in the healthcare curriculum, especially in physiotherapy programmes, where students’ knowledge of RCT characteristics should be integrated early to ensure the transfer of EBP to provide the best care. In the future, this initiative should potentially be considered by lecturers, educational research promoters and stakeholders involved in UR programmes.

### Electronic supplementary material

Below is the link to the electronic supplementary material.

Supplementary Material 1

## Data Availability

The datasets used and/or analysed during the current study are available from the corresponding author on reasonable request.

## List of Abbreviations

EBP: Evidence-based practice
UR: Undergraduate research
RCT: Randomised controlled trial
