# Supplementary material for: Undergraduate research implementation in physiotherapy: a hands-on and real experience of a randomised controlled trial
Source: BMC Med Educ. 2023 Oct 6;23:736. doi: 10.1186/s12909-023-04716-0 (PMC10559614; doi:10.1186/s12909-023-04716-0)
Supplement: Supplementary file 1 — Supplementary Material 1 [file 12909_2023_4716_MOESM1_ESM.docx]

**Supplementary material A**

***Procedures related to RCT***

**Study design and registration**

This RCT was designed following the Consolidated Standard of Reporting Trials for Controlled Studies (CONSORT) statement, registered with the Australian New Zealand Clinical Registry (ACTRN12622000263796p, 14/02/2022) and approved by the Research Ethics Committee of the University of Deusto (ETK-21/21-22). None of the lecturers involved had any commercial relationship or conflict of interest with the private company that owned the device tested. All participants provided a consent form, and the study was undertaken according to the Helsinki declaration.

**Participants**

One hundred and fourteen students between 17 and 30 years of age were invited to participate and informed about the blinded RCT including possible effects and demands of the intervention. Exclusion criteria were pregnancy, and the use of a cochlear implant or a pacemaker. Twelve declined to participate and 102 students were finally included in the study.

**Methods**

Participants were randomly allocated to three groups (intervention, placebo, and control) directly after baseline assessment, using a computer-generated random sequence table. Students allocated into the intervention group wore a superficial neuromodulation device (© 2020 Irmoki). The device consists of 4 wireless receptors to be placed on the distal third of the limbs by means of gloves and anklets and controlled through Bluetooth technology by a smartphone app (Figure 1). The app remotely activates the device which emits rectangular biphasic and monophasic galvanic electrical impulses of very low frequency (0.5-14 Hz) coordinated through 28 electrodes that aim to modulate the autonomic nervous system and thus, generate a beneficial effect on general well-being: sleep, recovery, fatigue, stress, pain, and performance. Students in the intervention group wore the device while they remained seated at rest following lectures at University. This intervention was provided for three hours per week (divided into two days) and for eight weeks. Students in the placebo group wore the device in the same way as the intervention group, but without being active (light was switched on but the device did not emit the electrical current). Students in the placebo and intervention groups, as well as two of the four lecturers who delivered the devices to the students were blinded. The control group did not receive any intervention.

**Data collection and analysis**

Sociodemographic data were collected at baseline. Outcomes were gathered by means of an online questionnaire at baseline and after weeks 2, 4, 6, and 8. The questionnaire included five questions assessing the following domains: fatigue, sleep quality, muscular soreness (DOMS), stress level, and mood. Participants had to rate their status with a Likert scale, where five was the best and one was the worst. In addition, participants in the intervention and placebo groups were asked about: a) their feelings and expectations towards the intervention (sham for the placebo group); b) potential alterations of the sleep, urination, relaxation, and nervous conditions; and c) the extent to which the intervention had fulfilled their expectations. Students designed and executed the data analysis under the supervision of a lecturer, utilizing knowledge and skills acquired earlier in the first semester in another subject (“Biostatistics”).

**Supplementary material B.**

**Parts of the questionnaire collected several outcomes focused on four dimensions:**

1. **Dimension of the integration of research knowledge related to the theoretical contents**

| **Question:**  **According to the subject syllabus, how did the RCT implementation affect the learning process related to the theoretical contents?** | | | | | |
| --- | --- | --- | --- | --- | --- |
|  | **Very negative effect** | **Negative effect** | **No effect** | **Positive effect** | **Very positive effect** |
| Ethical aspects: ethical committee & informed consent |  |  |  |  |  |
| Evidence-based medicine and scientific method |  |  |  |  |  |
| Scientific article: structure of an article |  |  |  |  |  |
| Publication: journals, impact factor and peer review process |  |  |  |  |  |
| Systematic review |  |  |  |  |  |
| PICO search strategy |  |  |  |  |  |
| Research designs: types of study designs |  |  |  |  |  |
| Random errors and methodological biases |  |  |  |  |  |
| Measurement concepts: reliability and validity |  |  |  |  |  |
| Placebo |  |  |  |  |  |

1. **Dimension of the development of research competences**

| **Question:**  **In terms of the competencies developed in the subject, how did the RCT implementation affect the acquisition and development of the competencies?** | | | | | |
| --- | --- | --- | --- | --- | --- |
|  | **Very negative effect** | **Negative effect** | **No effect** | **Positive effect** | **Very positive effect** |
| GENERAL COMPETENCE: To update one's own learning, reflecting on one's own knowledge and way of learning, questioning the usual ways of acting and questioning the issues with a critical perspective. |  |  |  |  |  |
| SPECIFIC COMPETENCE 1. Identify and interpret the main methodological concepts in research to develop critical opinions, based on evidence, of the different areas of physical therapy. |  |  |  |  |  |
| SPECIFIC COMPETENCE 2. Assess the methodological quality and results of studies and/or research projects in the scientific field to develop critical capacity. |  |  |  |  |  |
| SPECIFIC COMPETENCE 3. Know and apply new and previous knowledge of biostatistics, bibliographic search to contrast and question the current scientific evidence of the main physiotherapy treatments of the different areas of action by writing a scientific article in small working groups. |  |  |  |  |  |

1. **Dimension of self-efficacy about the skills trained**

| **Questions**: | Not at all | Very little | Little | Enough | Quite a lot | Very much | Excellent |
| --- | --- | --- | --- | --- | --- | --- | --- |
| How competent do you feel in understanding the design of a study? |  |  |  |  |  |  |  |
| How competent do you feel in understanding the methodological quality of a study and identifying the biases or limitations of a study? |  |  |  |  |  |  |  |
| How competent do you feel in correctly designing a randomized clinical trial? |  |  |  |  |  |  |  |
| How competent do you feel in understanding and explaining the meaning of the placebo effect? |  |  |  |  |  |  |  |
| How competent do you feel in interpreting the results of a study? |  |  |  |  |  |  |  |

1. **Dimension of The RCT procedures**

| **Question:**  **Related to some procedural aspects of the RCT implementation…** | | | | | |
| --- | --- | --- | --- | --- | --- |
|  | Strongly disagree | Disagree | Agree | Strongly agree | Completely agree |
| The objectives of the RCT implementation have been well defined. |  |  |  |  |  |
| The methodology of the RCT implementation has been adequately framed in the subject. |  |  |  |  |  |
| The execution of RCT has been interesting and dynamic. |  |  |  |  |  |
| With RCT implementation I felt that I have participated more actively in the subject. |  |  |  |  |  |
| It has been a good experience to share the RCT implementation with my classmates. |  |  |  |  |  |
| What I have learned with this RCT implementation can be useful for other subjects. |  |  |  |  |  |
| This activity has contributed to my learning of the subject. |  |  |  |  |  |
| You learn more actively with this activity than with other more traditional activities. |  |  |  |  |  |
| The involvement and organization of the teaching team has been adequate and structured. |  |  |  |  |  |
| The amount of time we have dedicated to the IRMOKI project has been the right one. |  |  |  |  |  |

1. **General opinion questionnaire of students and teachers**

| **Questions:** |
| --- |
| As student, what aspect of the RCT implementation would you highlight in your learning process?  As teacher, what aspect of the RCT implementation would you highlight in your teaching process? |
